# Foraging behaviour and ecology of transient killer whales within a deep submarine canyon system

**DOI:** 10.1371/journal.pone.0299291

**Published:** 2024-03-20

**Authors:** Josh D. McInnes, Kevin M. Lester, Lawrence M. Dill, Chelsea R. Mathieson, Peggy J. West-Stap, Stephanie L. Marcos, Andrew W. Trites

**Affiliations:** 1 Institute for the Oceans and Fisheries, Marine Mammal Research Unit, University of British Columbia, Vancouver, Canada; 2 Pacific WildLife Foundation, Port Moody, BC, Canada; 3 Oceanic Ecology Research Group, Monterey Bay, California, United States of America; 4 Department of Biological Sciences, Evolutionary and Behavioural Ecology Research Group, Simon Fraser University, Burnaby, Canada; 5 School of Resource and Environmental Management, Simon Fraser University, Burnaby, Canada; 6 Marine Life Studies, Moss Landing, California, United States of America; Hawaii Pacific University, UNITED STATES

## Abstract

Transient killer whales have been documented hunting marine mammals across a variety of habitats. However, relatively little has been reported about their predatory behaviours near deep submarine canyons and oceanic environments. We used a long-term database of sightings and encounters with these predators in and around the Monterey Submarine Canyon, California to describe foraging behaviour, diet, seasonal occurrence, and habitat use patterns. Transient killer whales belonging to the outer coast subpopulation were observed within the study area 261 times from 2006–2021. Occurrences, behaviours, and group sizes all varied seasonally, with more encounters occurring in the spring as grey whales migrated northward from their breeding and calving lagoons in Mexico (March-May). Groups of killer whales foraged exclusively in open water, with individuals within the groups following the contours of the submarine canyon as they searched for prey. Focal follows revealed that killer whales spent 51% of their time searching for prey (26% of their time along the shelf-break and upper slope of the canyon, and 25% in open water). The remainder of their time was spent pursuing prey (10%), feeding (23%), travelling (9%), socializing (6%), and resting (1%). Prey species during 87 observed predation events included California sea lions, grey whale calves, northern elephant seals, minke whales, common dolphins, Pacific white-sided dolphins, Dall’s porpoise, harbour porpoise, harbour seals, and sea birds. The calculated kill rates (based on 270 hours of observing 50 predation events) were 0.26 California sea lions per killer whale over 24 hours, 0.11 grey whale calves, and 0.15 for all remaining prey species combined. These behavioural observations provide insights into predator-prey interactions among apex predators over submarine canyons and deep pelagic environments.

## Introduction

Transient killer whales are one of at least three ecotypes (transients, residents, and offshores) of *Orcinus orca* inhabiting the North Pacific Ocean, and are the only ecotype within this region that specializes in feeding on marine mammals [1,2]. Transient killer whales distributed along the west coast of North America from Southeast Alaska to Southern California belong to the genetically distinct west coast population. Members within this population appear to comprise two discrete clusters that occasionally associate with each other, but have heterogenous distributions and habitat use patterns—the *inner coast* and *outer coast* transient subpopulations [3–5].

Individuals within the inner coast subpopulation primarily inhabit the relatively shallow coastal inland waters of British Columbia, Washington State, and Southeast Alaska where they feed on pinnipeds and small cetaceans in open straits and bays, around islets and kelp strewn reefs, and near dense ice filled waters of tide water glaciers [1,2,6–8]. These shallow habitats are characterized by strong tidal currents and high bottom relief topography. Whales that occur here appear to be drawn by the reproductive cycle of harbor seals (*Phoca vitulina*) and the seasonal movements of Steller (*Eumetopias jubatus*) and California (*Zalophus californianus*) sea lions [1,2,9].

Along the coast of Oregon, whales belonging to the inner coast subpopulation have been documented feeding on harbor seals, Steller and California sea lions, and grey whale calves (*Eschrichtius robustus*) around unsheltered reefs and islets in relatively shallow, high energy sandy shoreline areas, and near river mouths, saltwater estuaries, and mudflats [4]. Most sightings occur during the harbour seal pupping period from April through June [10].

In contrast to the nearshore habitats, members of the outer coast transient killer whale subpopulation have been documented hunting marine mammals in open ocean and deep pelagic habitats such as the continental shelf-break and deep submarine canyons—with increased observations occurring off the California coast [3,11]. However, accounts from deeper offshore waters have been sporadic and opportunistic, and have primarily yielded qualitative data concerning predatory behaviours, seasonal occurrences, social dynamics, and habitat use [12]. Dedicated studies have not been undertaken to quantify behavioural and ecological aspects of these apex predators in such deep-water ecosystems.

**Fig 1:**
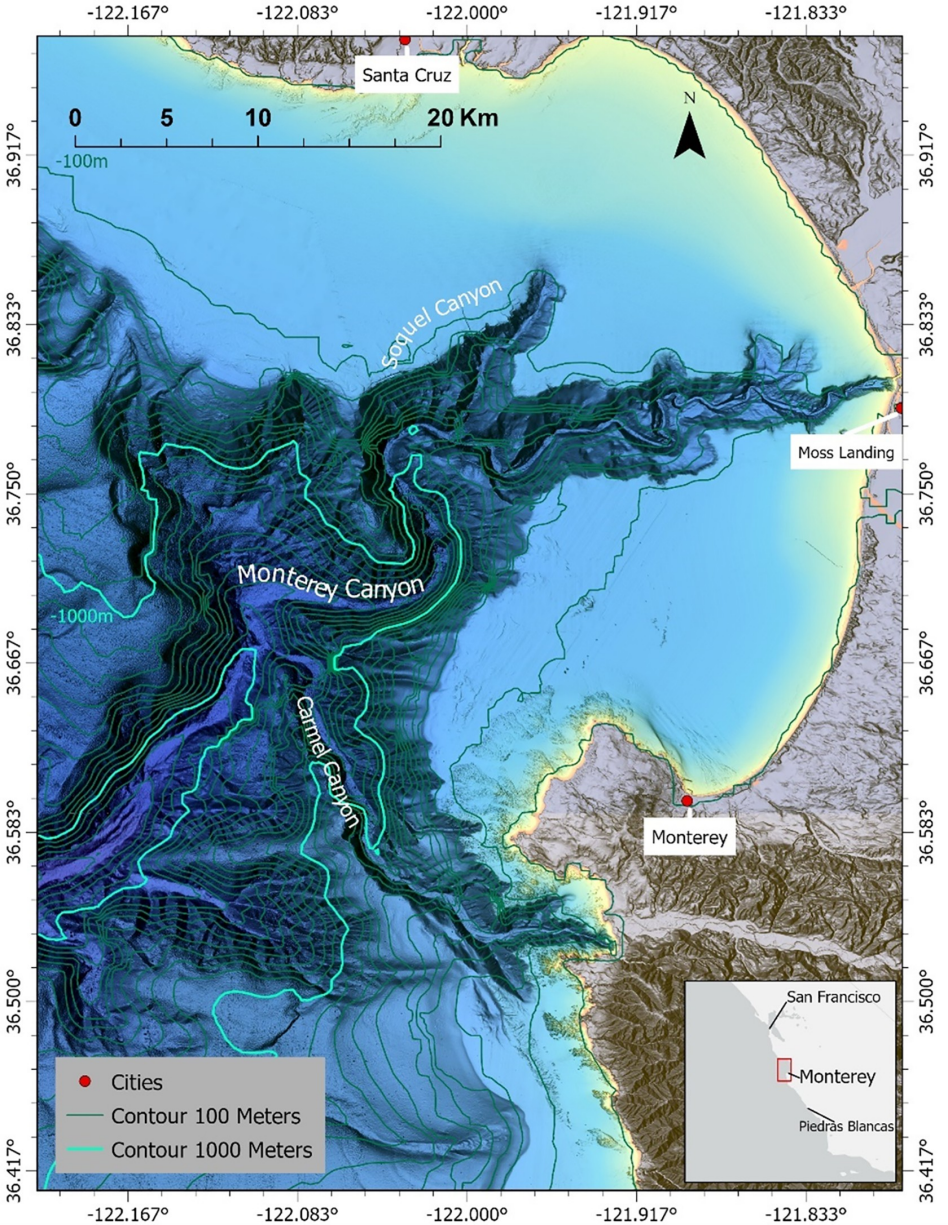
Study area and submarine canyon system of Monterey Bay, California. Base map Reprinted from NCEI under a CC BY license, with permission from NOAA, original copyright 2022.

The goal of our study was to describe the predatory behaviour of transient killer whales in a deep-water habitat. Thus, we used a long-term dataset of observations to quantify the seasonal occurrence patterns, behaviours, diets, and habitat use of these whales in and around the Monterey Submarine Canyon, in Monterey Bay, California (Fig 1). This canyon is the largest of its kind to transect the narrow continental shelf off the Central California coast—and is considered to be a ‘biological hotspot’ for marine mammals due to complex oceanographic processes and high habitat heterogeneity [13–15].

The observations we made of transient killer whales in the Monterey Submarine Canyon System contribute to understanding the behavioural ecology of these apex predators and their interactions with prey in the North Pacific. Most notably they document the adaptations of a distinct outer coast subpopulation of transient killer whales that employ different foraging strategies to exploit prey in habitat that differ significantly from the coastal shallow waters where most studies on transients have occurred to date. Our observations also contribute to understanding the potential of these predators to exert strong top-down effects in marine food webs in California waters, and the impact they may have on potentially vulnerable prey populations, such as eastern North Pacific grey whales. Lastly, our findings contribute to marine mammal stock assessments in the North Pacific, and can be used to define the habitat needs of transient killer whales along the central coast of California.

## Results

**Fig 2:**
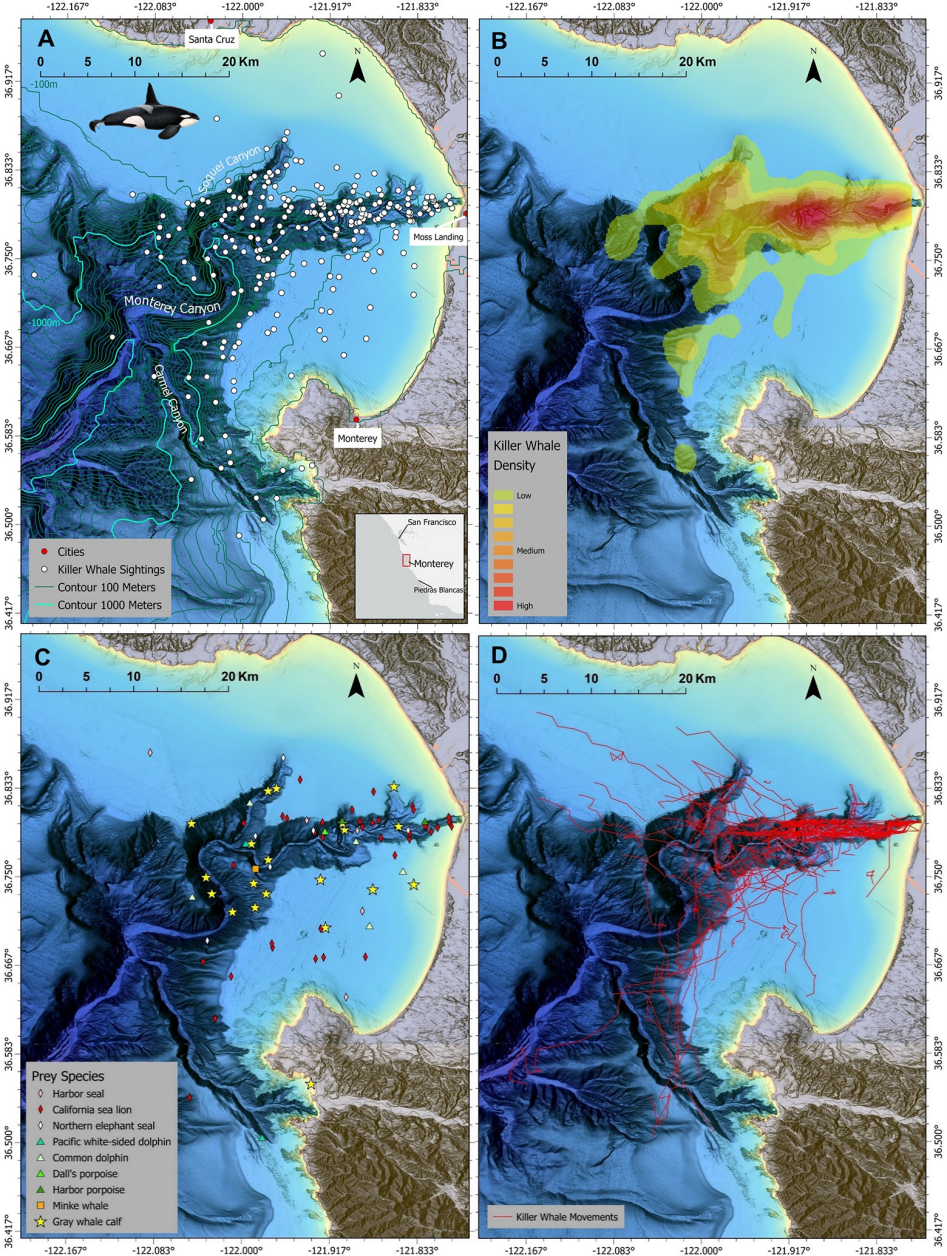
Geographic locations of recorded observations of transient killer whales in Monterey Bay, California. **(A)** Distribution of recorded observations (sightings and surveys) where each white circle represents an independent observation of the presence of one or more whales **(n = 261 recorded observations; 2006–2021)**; and the inserted map indicates the location of the main study area off the central coast of California. **(B)** Relative spatial densities of transient killer whales based on recorded observations (panel A) showing hotspots over the Monterey Submarine Canyon System. **(C)** Locations where predation by transient killer whales occurred on 9 species of marine mammals **(n = 87; 2006–2021). (D)** Tracks of the research vessel as it followed groups of transient killer whales **(n = 100 focal follows; 2006–2018)** that were typically <50 m from the research vessel. Data are visualized using kernel density against NOAA 10-meter resolution bathymetry. Base maps reprinted from NCEI under a CC BY license, with permission from NOAA, original copyright 2022.

Transient killer whales were observed within the study area on 261 occasions from 2006–2021 (Fig 2A). During this period, we photo-identified 183 different individual whales, comprising 51 matrilineal groups and 16 solitary males. All were identified as outer coast transient killer whales [4,11]. Dedicated marine mammal vessel-based surveys occurred on 559 days from 2006–2018. During this period transient killer whales were encountered 100 times within Monterey Bay and were followed for 1324.6 km ($\bar{x}$ ± SE = 16.4 ± 1.21 km, n = 100 focal follows) (Fig 2D)—resulting in ~270 hours of behavioural observation.

**Fig 3:**
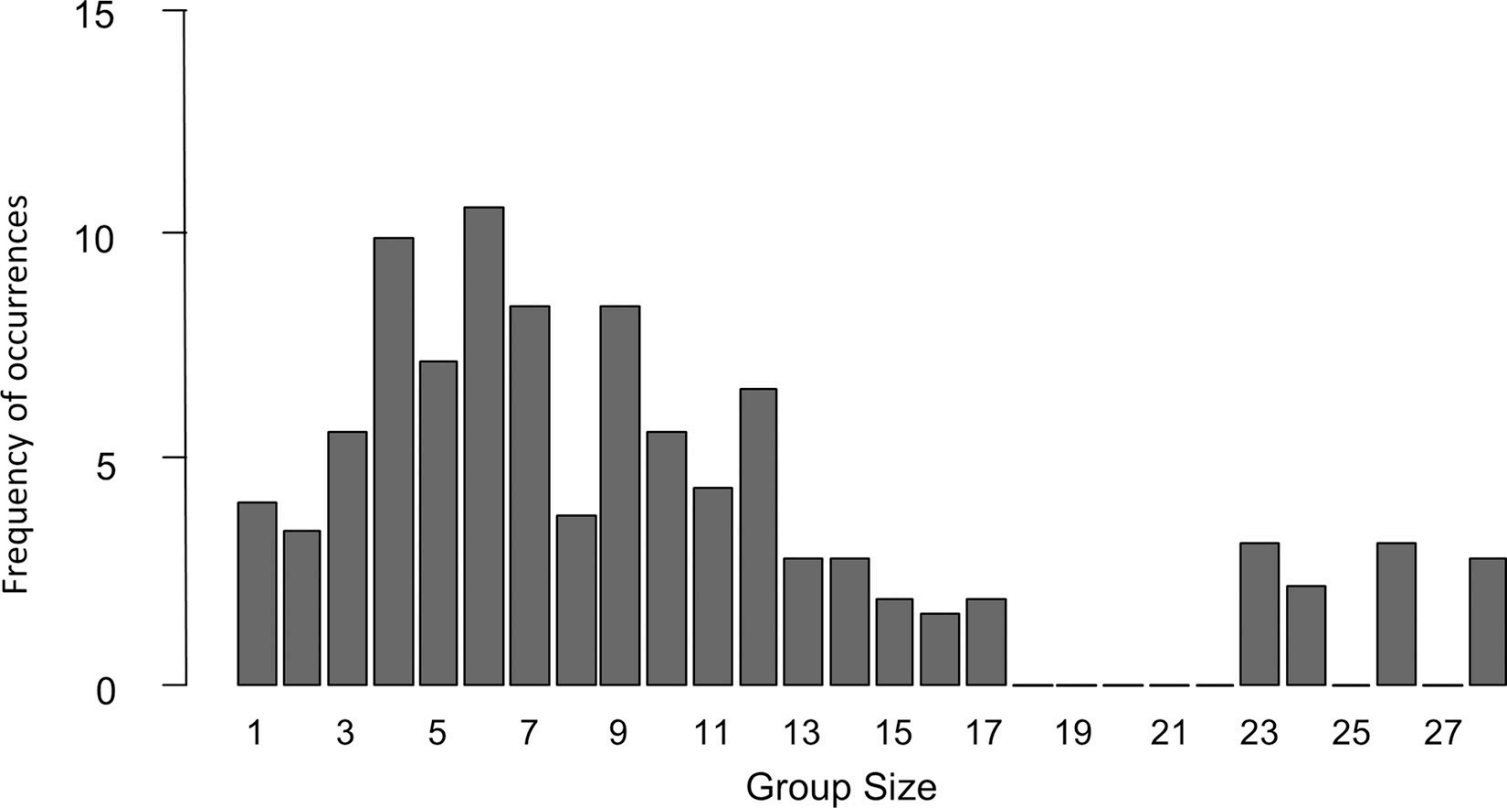
Group sizes of transient killer whales in Monterey Bay, California from 2014–2021. Group sizes ranged from 1–28 individuals, with the most frequently observed group size being 6 whales (mode = 6, $\bar{x}$ ± SE = 9.71 ± 0.38, n = 323 occurrences).

Whale watch sighting data were collected from 2014–2021, with ecotours operating everyday (weather permitting) throughout the year. The frequency with which different photo-identified matrilineal groups and solitary males visited the study area during this period (excluding resightings within a 4-day period) totaled 323 independent occurrences. Transient group size during this time ranged from 1–28 whales, with the most frequently observed group size being 6 whales (mode = 6, $\bar{x}$ ± SE = 9.71 ± 0.38, n = 323 occurrences; 2014–2021) (Fig 3).

### Seasonal occurrence

**Fig 4:**
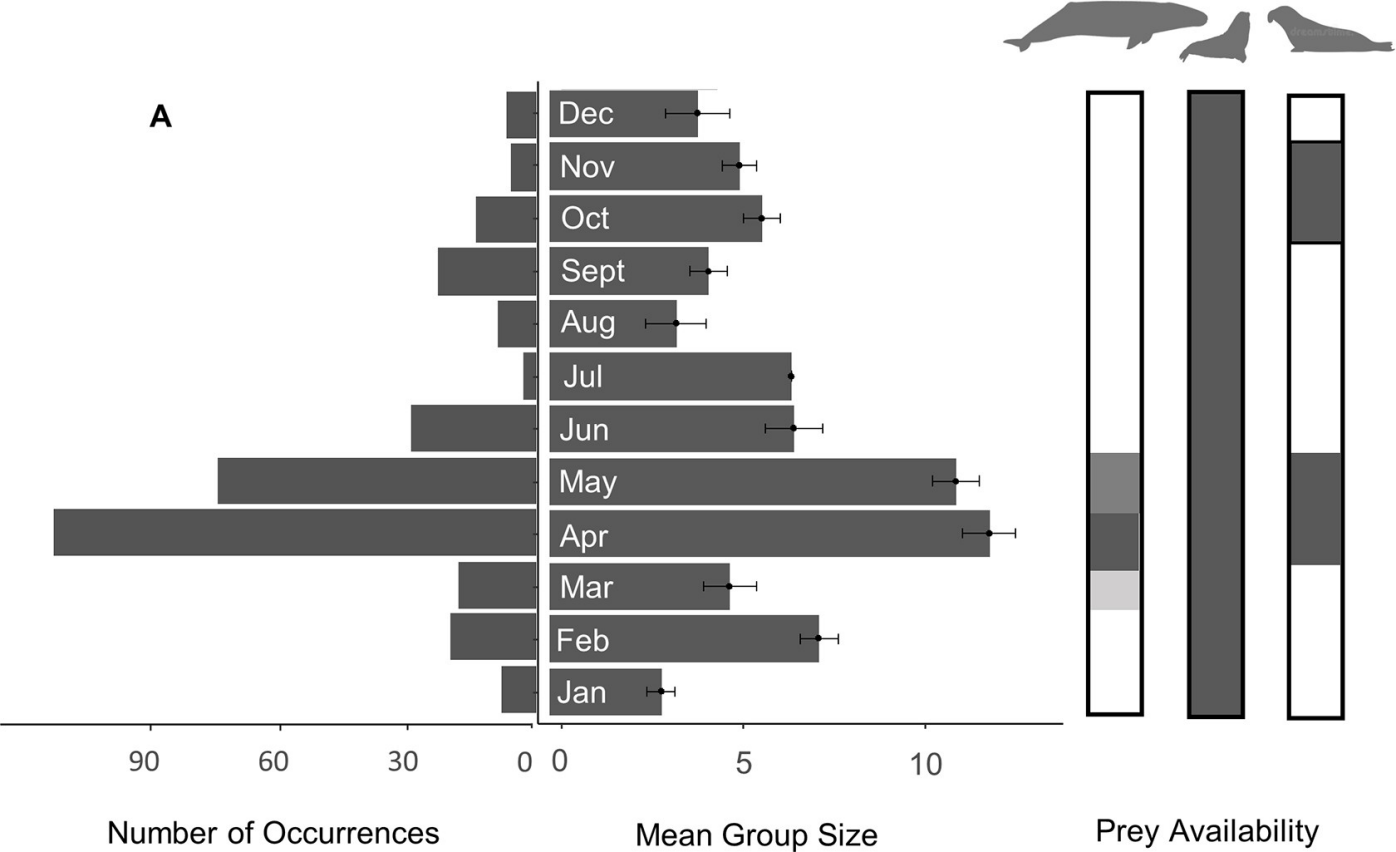
Seasonal presence of transient killer whales relative to the availability of prey species. **(A)** Number of occurrences of transient killer whale by month in Monterey Bay, California, 2014–2021 (n = 323 sightings); and **(B)**, mean group sizes with standard error bars relative to **(C)** seasonal presence (grey shading) of California sea lions, grey whale calves, and northern elephant seals. Each occurrence is a specific matrilineal transient family, and prey phenologies are adapted from [16–18].

Transient killer whales occurred in Monterey Bay in all months of the year from 2014–2021. However, we found significant differences in occurrence among months (Kruskal-Wallis χ^2^ = 30.8, df = 11, *p* < 0.001) (Fig 4A)—particularly between the seasons of March–May and June–February (Student’s t test = 2.21, df = 10, *p* = 0.026). Transient killer whales occurred most frequently in April and May, which was followed by a sharp decrease in occurrence in June–August, and a slight increase from September–November (Fig 4B).

**Fig 5:**
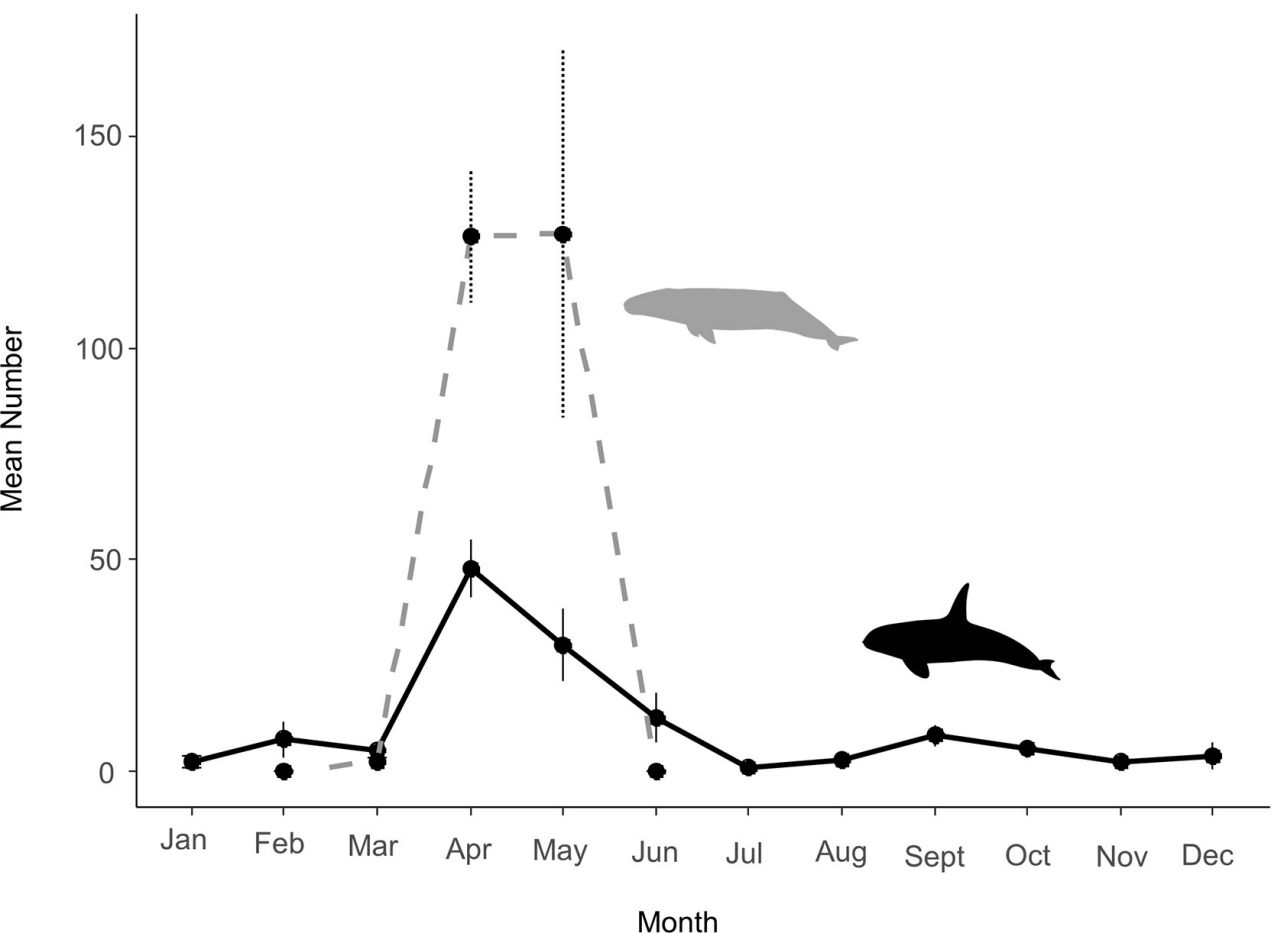
Mean monthly numbers of transient killer whales and grey whale calves. Numbers (± SE) of individually identified transient killer whales were observed in Monterey Bay (solid black line) and mean numbers of grey whale calves were counted at the Piedras Blancas field station off the central coast of California (dashed grey line) from 2014–2021. Grey whale calf census data from [18–20].

Seasonal distribution patterns aligned with the annual cycles of three prey species off the central coast of California—California sea lions, which are present in Monterey Bay throughout the year (peaking in September through November); eastern North Pacific grey whale mothers with calves that migrate north along the coast (peaking in April and May); and northern elephant seals (*Mirounga angustirostris*), both adult females that haul out during a spring molt (peaking in April and May), and juvenile elephant seals that haul out during the fall (peaking in October and November) (Fig 4C). The largest numbers of different individual photo-identified killer whales occurred in April-May, concurrent with the highest counts of grey whale calves, but also coinciding with the peak in adult female elephant seals that haul out along the coast (Fig 5). Group size during this time (from March–May) was significantly larger ($\bar{x}$ ± SE = 11.7 ± 0.51) than from June–February ($\bar{x}$ ± SE = 5.8 ± 0.35) (Mann-Whitney U test, W = 5615.5, *p* < 0.001).

### Habitat use

Encounters and sightings of transient killer whales occurred throughout Monterey Bay from 2006–2021, with observations occurring in water depths averaging 340.4 m (SE = 18.75 m, range = 6–1645 m, n = 261 observations) at an average distance from the nearest shore of 9.6 km (SE = 0.3 km, range 0.2–25.5 km, n = 261 observations). However, observations were highest along the continental shelf break and slope of the primary central channel of the Monterey Submarine Canyon (Fig 2B) about 6.9 km ($\bar{x}$) from shore (SE = 0.35 km, range = 1.2–12.7 km, n = 261 observations) in water depths averaging 250.2 m (SE = 15.9 m, range = 36–594 m, n = 261 observations).

Predation by transient killer whales on marine mammals occurred throughout the study area (Fig 2C) at an average distance of 8.8 km from the nearest shore (SE = 0.52 km, range = 2.2–18.5 km, n = 87 predation events) over average water depths of 313.8 m (SE = 33.4 m, range = 6–1374.1 m, n = 87). Among the three most commonly observed prey species (California sea lions, grey whale calves, and northern elephant seals), water depths and geospatial locations of successful predation events differed significantly (Kruskal-Wallis χ^2^ = 7.48, df = 2, *p* = 0.02).

We observed 42 predations on California sea lions, which occurred in both shallow continental shelf waters and in deeper waters along the continental shelf break of the submarine canyon. On average, sea lions were preyed upon 7.1 km ($\bar{x}$) from shore (SE = 7.3 km, range = 0.78–18.5 km, n = 41 predation events) in water averaging 220.9 m deep ($\bar{x}$ ± SE = 220.9 ± 31.1 m, range = 6–770.4 m, n = 41). In comparison, 19 successful predations on grey whale calves occurred in significantly deeper water ($\bar{x}$ = 419.4 m, SE = 89.7 m, range = 6–1374.1 m, n = 19) (Mann-Whitney U test, W = 250, *p* = 0.035). One additional predatory interaction occurred on a grey whale calf close to shore in the shallow waters (~6 m) of Carmel Bay. However, the injured calf escaped with its mother into a nearby kelp bed, and the killer whales abandoned the hunt.

In contrast to California sea lions, attacks on northern elephant seals were observed in significantly deeper waters (Mann-Whitney U test, W = 42, *p* = 0.008), with all events occurring over the central channel of the canyon in water depths averaging 515.7 m (SE = 171.2 m, range = 114.4–789.6 m; n = 6 predation events). However, there was no significant difference between the water depths where predation of grey whale calves and northern elephant seals occurred (Mann-Whitney U test, W = 46, *p* = 0.51). The remaining prey species (dolphins, porpoise, minke whales and harbour seals) represented < 10% of observed predations and therefore sample sizes were too small for geospatial statistical analysis.

### Behavioural ecology

Behavioural data collected during 100 focal follows from 2006–2018 totalling ~270 hours of observation, and lasting anywhere from 10 minutes to 9.5 hours, showed that whales spent an average of 1% of their time each day resting, 6% socializing, 9% travelling, and 84% foraging (Table 1).

**Table 1:** Activity budget of transient killer whales based on 270 hours of behavioural observations.

Behaviour	Percentage of time for each category	Percentage of time for each subcategory
Foraging	84.16	
Shelf break/canyon foraging		26.13
Open water foraging Prey pursuit		24.68 10.37
Feeding		22.98
Travelling	9.34	
Socializing	5.54	
Resting	0.97	

(see Table 5 for description of each activity state).

#### Foraging behaviour

**Fig 6:**
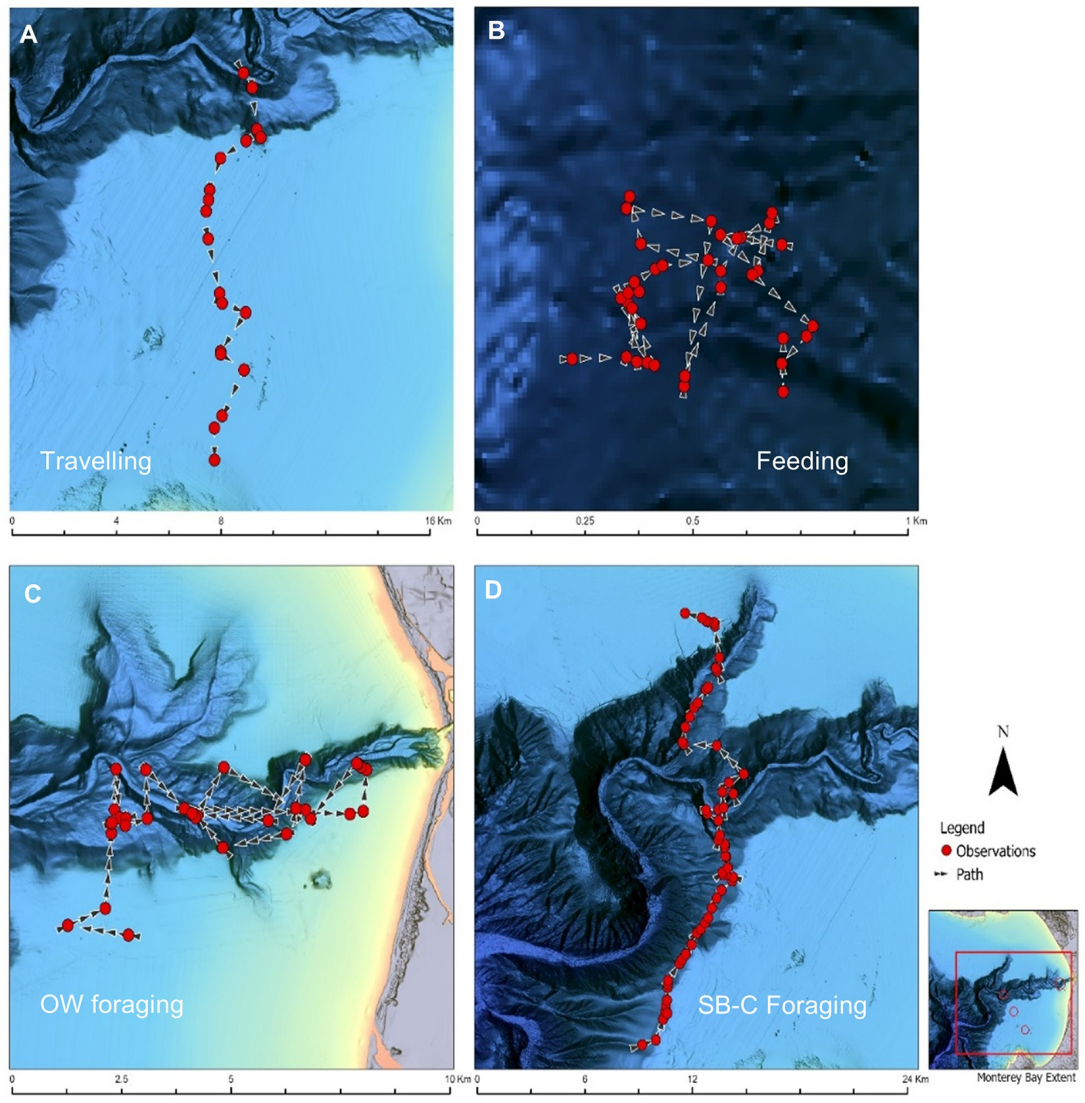
Detailed movements of transient killer whale groups during focal follow surveys, 2006–2018. These examples show n = 4 of 100 focal follows in Monterey Bay, California that is typical of **(A)** travelling, **(B)** feeding, **(C)** open water foraging, and **(D)** shelf break foraging. Groups of whales were usually followed within 50 m while recording whale behaviours and georeferenced data. Each red circle represents a georeferenced location of the research vessel when an accompanying transient killer whale group surfaced, and arrows indicated direction of movement. Note scale differences between panels. Base map reprinted from NCEI under a CC BY license, with permission from NOAA, original copyright 2022.

Foraging (including prey pursuit and feeding) accounted for 84% of the total time killer whales were observed (Table 1) and consisted of two types of behaviours—***canyon/shelf break foraging***, and ***open water foraging*** (Fig 6).

***Open water foraging*** always involved transient killer whales searching for prey in open water not always associated with the submarine canyon. Whales performed long asynchronous dives (>8 minutes), with individuals spread out up to 1 km apart and displaying erratic (zigzagging) movements with no cohesion in group movements (Fig 6C). Open water foraging comprised 25% of the overall time spent observing transient killer whales (Table 1), and observations of predation events using this foraging technique primarily involved small cetaceans and pinnipeds.

***Canyon/shelf break foraging*** typically involved searching for prey in a single or double front formation [21]. The whales would follow the contours of the submarine canyon (200–500 m isobaths) or they would cross over the deep central channel to explore adjacent areas along the canyon, with prolonged foraging in these areas often resulting in multiple successful predation events (Fig 6D). The distance between individual whales was usually less than 6 m, with members of the group conducting long synchronous dives (5–7 minutes) while moving in a consistent direction along the shelf break. Canyon/shelf break foraging encompassed 26% of the total time the whales were observed (Table 1), and most predation events involving grey whale calves (75%) occurred during this form of foraging.

**Fig 7:**
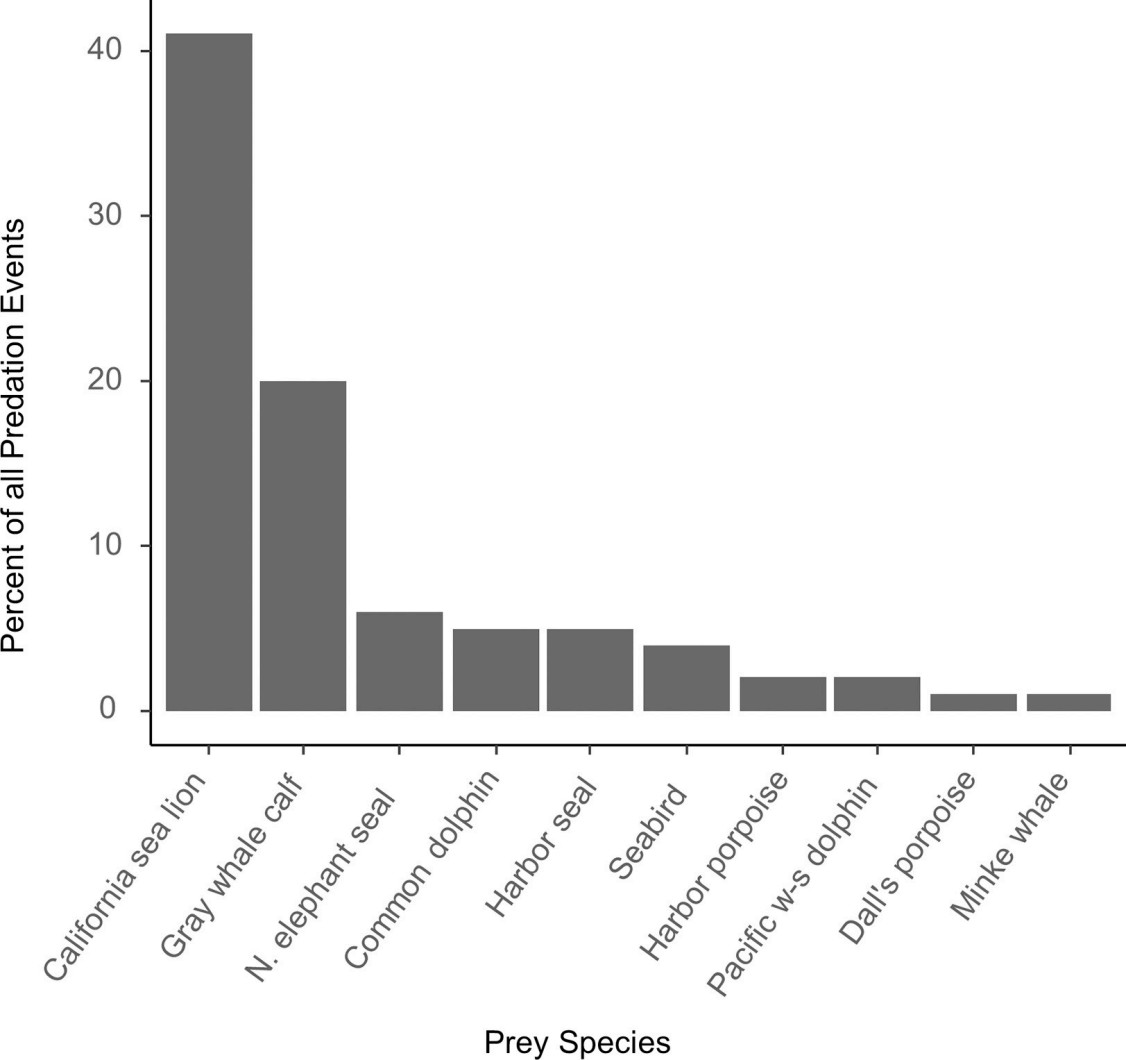
Frequency with which 10 species of prey were killed by transient killer whales during 87 predation events observed during focal follow surveys and reports by whale watchers in Monterey Bay, California 2006–2021. California sea lions and grey whale calves accounted for >60% of the kills. Seabird species are combined.

Of the 94 predation events recorded during vessel based focal follows and from whale watch reports (2006–2021), 87 involved prey that could be identified to species and were used in the analysis—of which 50 were identified during focal follows (used to calculate kill rates) and 37 were reported by whale watchers. In all, transients consumed 12 different species, including 6 species of cetacean, 3 species of pinniped, and 3 species of seabird (Fig 7). However, the majority of predation events involved California sea lions, grey whale calves, and elephant seals.

**Fig 8:**
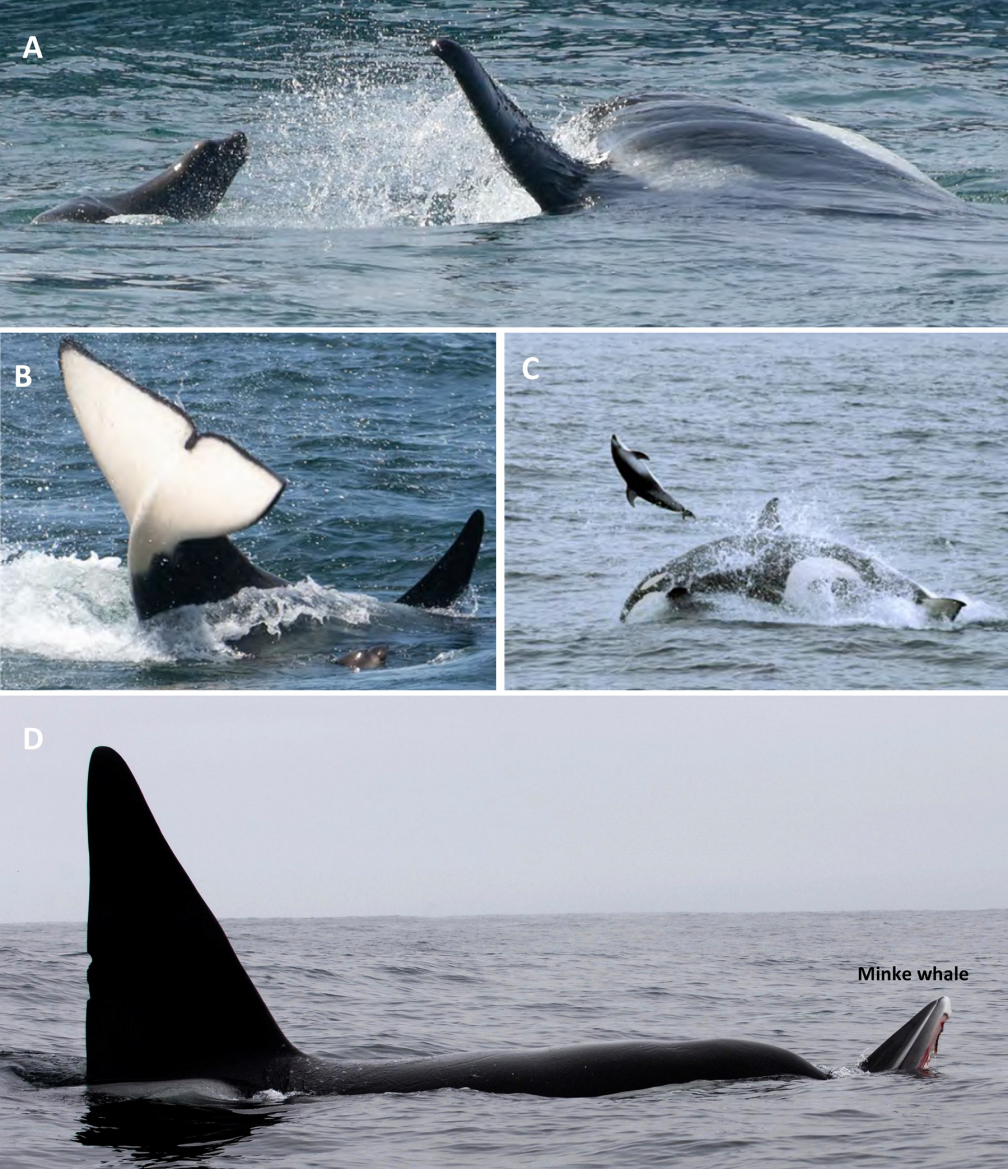
Predation events by transient killer whales on marine mammals in Monterey Bay, California. Prey include, **(A)** California sea lion being pursued, **(B)** juvenile northern elephant seal being hit by the tail of a transient, **(C)** Pacific white-sided dolphin being thrown through the air, **(D)** Adult male transient killer whale OCT004 grasping the rostrum of a minke whale. Photo credits: Chelsea Mathieson (A), Peggy West-Stap (B-D).

Prey handling made up 33% of the time transient killer whales were observed. Of this, *prey pursuit* (time observed encountering, attacking, and capturing prey) accounted for 10% of the observation time, and *feeding* (time from when the prey was killed to complete consumption or abandonment of the carcass) accounted for 23% of it (Table 1). The tactics used to pursue marine mammal prey were highly variable and dependent on species (Fig 8 and Table 2).

**Table 2:** Prey species targeted by transient killer whales and a description of predatory behaviour, number of attacks, and foraging group size.

Targeted species	Transient killer whale predatory behaviour	# of predation events	Group size
California sea lion	Coordinated prolonged hunt: whales surround and strike prey with their rostrums, sides, and flukes. Prey is often thrown into the air. Hunts may last >1 hour.	42	1–15 whales
Grey whale calf	Long coordinated hunts: prey pursued long distances; whales will try to separate female from calf; prey attacked dorsally near the blowhole area; prey rammed repeatedly; may remove large sections of tissue while prey is alive; prey movement hindered by whales biting and holding pectoral flippers, tail stock, and flukes; whales will breach onto the prey’s rostrum to try and drown it. Hunts may exceed 5 hours.	19	4–28 whales
Northern elephant seal	Short duration hunt: prey hit with whales’ flukes and rammed with rostrum; multiple whales surround prey, while one or two whales dive beneath prey; prey consumed beneath surface; large oil slick present. Hunts last < 20 minutes.	6	3–16 whales
Harbour seal	Short duration hunts: prey dragged beneath the surface; typically consumed quickly with only small bits of blubber at the surface. Hunts typically last < 5 minutes.	5	1–5 whales
Pacific white-sided dolphin	High speed coordinated chase: whales leap clear of the water while flanking prey from all sides to separate individuals from the larger school; prey hit from underneath and thrown into the air. Longer duration hunt than Dall’s porpoise, >15 minutes.	2	2–6 whales
Common dolphin	Similar hunting behaviour used for Pacific white-sided dolphins but may involve larger groups of whales and larger dolphin schools. Hunts >15 minutes.	5	5–11 whales
Harbor porpoise	Short duration hunt: whales typically circle and chase; prey thrown into the air or grasped in whales’ mouth and dragged beneath the surface; consumed quickly. Rib cage, lungs, and heart left at the surface. Hunts typically last < 5 minutes	2	1–6 whales
Dall’s porpoise	Short duration high-speed coordinated hunt: individual whales take turns chasing single prey; breaching high into the air; prey launched into the air by whale’s rostrum. Highly visible event.	1	8 whales
Minke whale	Long duration high-speed hunt: whales will surround prey from multiple sides; may remove large sections of tissue while prey is alive; prey movement hindered by whales biting and holding pectoral flippers, tail stock, and flukes; prey dragged beneath the surface; large oil slick present at surface.	1	9 whales
Seabirds	Prey chased at the surface; grabbed and consumed or played with and released debilitated. Typically involves young whales. Hunts last < 5 minutes but can be prolonged with young whales.	4	1–7

***California sea lions****—*Transients were observed preying on adult male California sea lions throughout the year, with a small peak in the number of predations occurring in October through November (42 of all 87 events identified to species, of which 25 of 50 occurred during focal follows). Killer whale group size during California sea lion predation events ranged from 1–15 whales (mode = 5, $\bar{x}$ = 5.9 whales) (Table 2). During sea lion hunts, adult male and female killer whales took part in the attacks, while juveniles and young calves primarily stayed at the periphery of the hunt (~30–50 m away). Solitary males were also observed killing and feeding on California sea lions (5/42). Predatory behaviour included multiple whales surrounding the sea lion and taking turns rushing in and ramming or striking it with their rostrums or flukes (Fig 8A). Whales were also observed grasping onto and throwing sea lions into the air. On closer inspection of an abandoned carcass of one sea lion, we noted the animal had sustained lacerations to the hind flippers, abdominal puncture wounds, and semi-elliptical abrasions. Once killed, members of the group divided and consumed parts of the carcass or carried the carcass in their mouths for various amounts of time. California sea lion hunts lasted from 18 minutes to 1.5 hours.

Anti-predator behaviour of California sea lions involved becoming highly vigilant, sometimes accompanied by loud vocalizations, and forming large groups (>50 sea lions) at the surface (*rafting behaviour*). Individual sea lions were also observed hiding by remaining motionless in mats of drifting kelp or near the hulls of whale watching vessels. During 3 encounters, California sea lions were observed swimming and diving in close association with humpback whales (*Megaptera novaeangliae*) while the killer whales were close by.

***Grey whale calves****—*Transient killer whales selectively preyed and fed on yearling grey whale calves accompanied by their mothers during 19 of the 87 predation events we identified to species (11 of 50 occurring during focal follows). All observed predation events on grey whale calves occurred during the months of April and May. During grey whale calf predation events, group size ranged from 4–28 whales (mode = 15, $\bar{x}$ = 13.1 whales) (Table 2).

**Fig 9:**
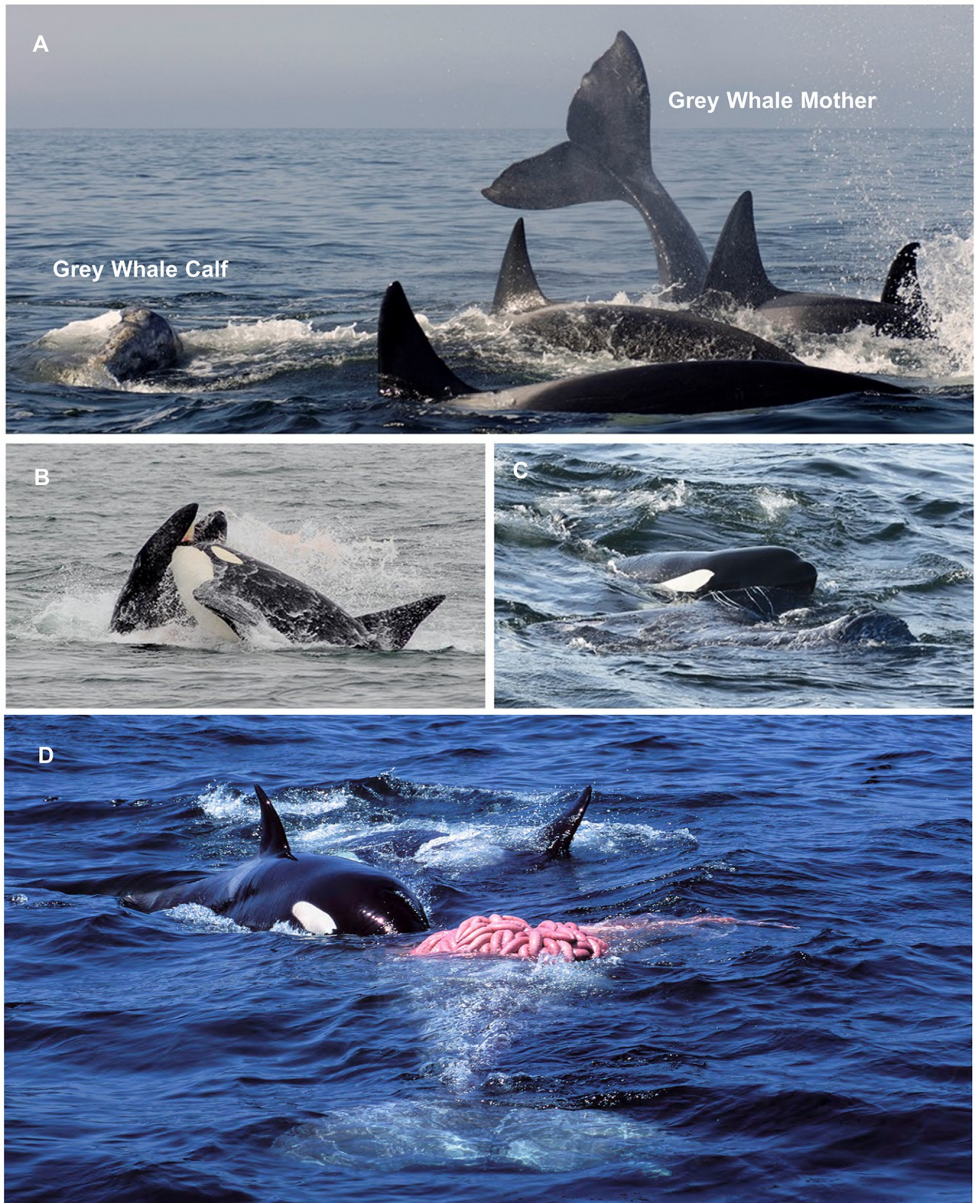
Predation of a grey whale calf by transient killer whales. Key moments include **(A)** the grey whale mother being separated from her calf, **(B)** the calf being rammed by an adult male transient, **(C)** before being submerged and drowned and **(D)** transient killer whales feeding on the grey whale calf lower jaw and tongue. Photo credits: Peggy West-Stap (A), Eric Austin-Yee (B), Stephanie Marcos (C), Peggy West-Stap (D).

Predatory behaviours employed to capture and kill grey whale calves varied among predation events, but all shared a few common traits (Fig 9). Most notably, the hunts were often initiated by adult females once grey whale mother-calf pairs were located. The adult female transients were also frequently accompanied by juveniles or young calves that dove and surfaced near the grey whales. As a group, the transients would then strategically herd or chase the female grey whale and her calf until the calf appeared to tire. Once tired, individual transients from the group would try to separate the mother from her calf by maneuvering their bodies between the pair or by dragging the calf away by grasping its flukes or pectoral fins (Fig 9A). Once separated from its mother, members of the group would try and incapacitate the calf by ramming the head and side regions and biting and removing large sections of tissue—often leaving the calf with rake marks, chunks of blubber missing, lacerated flukes and pectoral fins, and blood and oil in the water. Adult male killer whales were frequently observed using their rostrums to deliver blows to the head and sides of the calves (Fig 9B). Towards the end of the hunts, killer whales often appeared to kill the calves by leaping on top their blowholes to force them beneath the surface and drown them (Fig 9C). The recorded grey whale calf hunts lasted 1.6–5.3 hours (Table 2).

**Fig 10:**
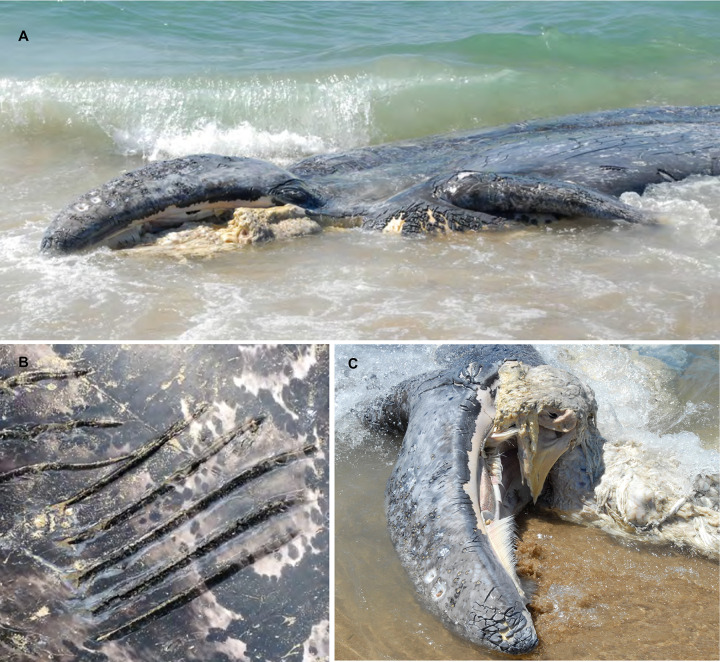
Deceased grey whale calf found stranded near Moss Landing, California on May 12, 2011. This calf was observed being attacked by a group of 10 transient killer whales the previous day in Monterey Bay. Note the missing lower jaw (A, C) and parallel cuts (B) made by the teeth of killer whales. Photo credit Peggy West-Stap.

When a grey whale calf was killed, killer whales from the group would dive for extended periods (>5 minutes) to feed on the carcass. Submerged grey whale calf carcases were easy to locate as they seeped oil that formed a slick at the surface. During periods when the calf carcass was visible at the surface, individual killer whales were seen grasping onto it and repeatedly rolling to dislodge chunks of blubber. At other times, individual killer whales would grasp onto the carcass and back away by pumping their flukes to dislodge chunks of tissue. Killer whales were also observed entering the calves’ mouth to consume the tongue and lower jaw (Fig 9D). Following one of these predation events, we examined the carcass that washed ashore the following day and noted that the calf’s lower jaw and tongue were completely missing (Fig 10). Periods of observed feeding typically lasted from 1.4–4.2 hours. However, one predation resulted in a group of 12 transients feeding for over 12 hours.

Anti-predator behaviour displayed by the grey whale mothers and calves involved making long dives and exhaling underwater with their blowholes barely exposed at the surface (snorkel behaviour) in an apparent effort to remain undetected [22]. Mother-calf pairs were also seen traveling with other adult grey whales (escorts) as they crossed the submarine canyon. When attacked, mothers rolled and thrashed their flukes from side to side and used their rostrums to physically ram individual killer whales. During four predation events, mothers positioned their calves on their backs away from the attack—in an apparent attempt to protect the calf’s vulnerable head and keep it above the water. During one predation event, a grey whale mother and her injured calf escaped a group of transients by taking refuge in a dense giant kelp forest (*Macrocystis pyrifera*) in shallow water (~6m) in Carmel Bay (Fig 2C). The killer whales abandoned this hunt and returned to deeper water.

***Northern elephant seals and harbor seals****—*We recorded transients attacking and feeding on juvenile and subadult male and female northern elephant seals during 6 of the 87 predation events on prey identified to species, of which 2 occurred during focal follows. Predation events on elephant seals occurred from May through December. Harbour seals (*Phoca vitulina*) were killed and consumed during 5 of the 87 predation events (all during focal follows). Group size during attacks on elephant seals ranged from 3–16 whales (mode = 5, $\bar{x}$ = 7.67 whales) and on harbour seals from 1–5 (mode = 2, $\bar{x}$ = 3.00 whales) (Table 2). Here, transients surrounded the seal and used their flukes to hit and stun it (Fig 8B). They would then grasp the seal’s appendages and vigorously shake or swing the seal back and forth. Hunts on elephant seals ranged from 10–20 minutes, and feeding was observed primarily at the surface for up to an hour. In comparison, harbour seals were killed quickly (<5 minutes) with consumption primarily occurring beneath the surface.

***Common dolphins and Pacific white-sided dolphins***—Common dolphins (*Delphius delphis*) were attacked and consumed during 5 of the 87 predation events (of which 2 occurred during focal follows), while Pacific white-sided dolphins (*Lagenorhynchus obliquidens*) were the subject of 2 of the predation events (1 during a focal follow). Group sizes during attacks on common dolphins ranged from 5–11 whales (mode = 6, $\bar{x}$ = 6.4 whales) and from 2–6 whales for Pacific white-sided dolphins (Table 2). Predatory behaviours were similar while hunting both delphinid species: hunts were highly coordinated, with transients separating individual dolphins by flanking a large school. This typically involved high speed swimming, with killer whales often leaping clear out of the water behind or into a school of dolphins. Once a subset or an individual dolphin was separated from the larger school, the transients would ram the dolphin from underneath, throwing it into the air, or grasp the dolphin crosswise in their mouths, dragging it beneath the surface (Fig 8C). The hunts we observed on dolphins lasted from 15 to 25 minutes.

***Dall’s porpoise and harbour porpoise***—A single Dall’s porpoise (*Phocoenoides dalli*) and 2 harbour porpoises (*Phocoena phocoena*) were attacked and consumed during the focal follows. A single group of 8 whales attacked the Dall’s porpoise, and groups of 1 and 6 whales participated in the 2 harbour porpoise predation events (Table 2). We observed markedly different predatory behaviours for both species. The Dall’s porpoise hunt involved a high energy and coordinated pursuit lasting 21 minutes. Individual killer whales took turns chasing the Dall’s porpoise by leaping high into the air behind it. Once caught, the whales rammed the Dall’s porpoise with their rostrums, throwing the porpoise into the air. In contrast, both harbour porpoise predation events were short, lasting <5 minutes, with both porpoises being grasped and dragged beneath the surface where prey handling was difficult to observe. However, the rib cage, lungs, and heart appeared at the surface following both harbour porpoise predation events.

***Minke whale***—During one focal follow, a group of 9 transient killer whales attacked, killed, and fed on a single minke whale (*Balaenoptera acutorostrata*) (Table 2). The attack involved a high-speed long-distance chase, involving multiple killer whales corralling the minke whale, while biting and removing chunks of blubber. Towards the end of the hunt, individual killer whales rammed and leapt on top of the minke’s rostrum—with one adult male killer whale grasping the rostrum and forcing the minke beneath the surface (Fig 8D). The entire hunt (pursuit and kill) lasted 56 minutes and feeding on the carcass lasted 1.5 hours.

***Seabirds—***We observed 4 attacks on seabirds during focal follows: on 2 common murres (*Uria aalge*), 1 rhinoceros auklet (*Cerorhinca monocerata*), and 1 northern fulmar (*Fulmarus glacialis*). Individual whales chased birds at or beneath the surface before grasping them in their mouths. Both common murres were killed and consumed, while the rhinoceros auklet and the northern fulmar were toyed with and released with debilitating injuries (i.e., broken wings, legs, and neck). All four predation events involved young juvenile killer whales and lasted <5 minutes.

### Kill rate

The daily kill rate (based on 270 hours of observation during 100 focal follows) was 0.26 California sea lions per transient killer whale per day, 0.11 grey whale calves, and 0.15 for all other prey pooled.

### Non-predatory behaviours

#### Resting behaviour

Resting accounted for 1% of the daily activity budget, during which time individual killer whales within a group were generally stationary (tight group formation) or moving slowly (<3 km/h), making little headway against surface currents. Members within a resting group were observed in close association (<2 m apart) and performed synchronous surfacing intervals with dives lasting between 4–6 minutes.

#### Travelling behaviour

Travel behaviour made up 9% of the total activity budget. During periods of travel, groups of whales typically swam in a line, abreast of one another in front or double front formation [21] in a consistent direction at speeds of 4–6 km/h and were not observed capturing or pursuing prey (Fig 6A). While travelling, individuals in a group typically swam less than 2–4 m from each other.

#### Interactions between transient killer whales and other species

We recorded non-predatory behaviour between transient killer whales and other marine mammals during 15 interactions—11 with humpback whales, 3 California sea lions and 1 involving a blue whale (*Balaenoptera musculus*). Interactions with humpback whales primarily occurred while transients pursued or fed on other marine mammal species. During these interactions, the humpbacks aggressively approached the killer whales while making vocal trumpet blows. Humpback whales were also observed positioning their bodies between killer whales and their targeted prey (i.e., California sea lions). Similar observations have been previously reported [23]. We never observed killer whales attacking humpbacks. The single blue whale interaction involved a group of 5 killer whales rapidly swimming towards and diving aggressively beneath the large mysticete. The blue whale responded by diving and slapping its fluke on the surface. Non-predatory interactions between transients and California sea lions often involved the whales passing by or diving beneath the sea lions without any change in behaviour to indicate they were interested in attacking them.

Transient killer whales were also observed interacting with non-marine mammal species. While foraging and during successful predation events, we observed several seabird species following groups of whales as they hunted, or were observed scavenging bits of prey at kill sites. Black-footed albatross (*Phoebastria nigripes*) and northern fulmars were the most commonly identified species, with birds following whales over large distances until a successful kill was made.

During two separate encounters involving successful predation of grey whale calves, we observed blue sharks (*Prionace glauca*) circling just beneath the surface roughly 30 m away from feeding whales. However, the sharks were never observed scavenging from the floating grey whale calf carcass while the transients were present. Lastly, on two separate encounters, we observed transients harassing an unidentified Pacific salmon species (*Oncorhynchus* sp.) and an ocean sunfish (*Mola mola*) at the waters surface, but no feeding was observed.

#### Social behaviour

Socializing accounted for 6% of the daily activity budget. When socializing, whales engaged in aerial displays, spy hopping, tail lobbing, pectoral fin slapping, and interactions between individuals (i.e., whales chasing each other, penile extrusion, etc.). Social behaviours primarily occurred after a successful predation event. However, we also observed social behaviour co-occurring with feeding behaviours (i.e., whales carrying parts of prey for extended periods), while other members interacted socially.

During the spring, large social aggregations of different matrilineal groups frequently formed after successful kills of grey whale calves. After a hunting-group successfully dispatched a grey whale calf, other groups of transients not involved in the hunt would often arrive from offshore waters to feed and socialize. This was observed in 6 of 11 grey whale calf predation events during focal follows. Periods of socializing lasted from 20 minutes to 2 hours.

## Discussion

Our observations of the diet, foraging behaviours, and seasonal occurrence in Monterey Bay suggest that the ecology of the outer coast transient killer whales that exploit deep submarine canyons is unique from that of the inner coast transients that feed on marine mammals in the nearshore and relatively shallow waters of British Columbia, Washington State, and Southeast Alaska. The subpopulation of transient killer whales we observed in Monterey Bay preferentially stayed in open, deep water, and primarily fed on seasonally available California sea lions, grey whale calves, and northern elephant seals. They exhibited specialized hunting techniques that differ from those used to capture marine mammals in shallow near-shore waters associated with reefs, rocky outcroppings, and islets.

### Seasonal occurrence

Outer coast transient killer whales were seen throughout the year in Monterey Bay and in all years of study. However, their presence changed seasonally, most likely a result of changes in the seasonal availability of marine mammal prey, as has been suggested for transient populations in other areas of the North Pacific [1,2,9,24]. Most notably, the peak in killer whale numbers in Monterey Bay between April and May coincides with the northward migration of adult female grey whales with their recently born calves [18–20,25]. This is also when adult female northern elephant seals return to moult [26]. The smaller secondary peak in killer whale numbers in the fall coincides with the return of juvenile northern elephant seals [26] and the northward dispersal of adult male California sea lions from their breeding islands [27]. Similar seasonal peaks in killer whale presence have also been noted in adjacent waters of the Farallon Islands (California) during the spring and fall [28]. Some of the killer whales that frequent the Farallon Islands are also seen in Monterey Bay, suggesting some transients may exhibit localized movements related to seasonal prey availability [4].

During the spring of each year, adult grey whale mothers and calves migrate northward from subtropical nursery lagoons of Baja California, Mexico Sur, to their feeding grounds in temperate and polar seas along the Pacific Coast and Bering Sea [18]. Grey whale mother-calf pairs typically arrive off the central coast of California during late March, with the largest numbers occurring from April through early May [20]. Also around this time, adult female elephant seals arrive off the California Coast from offshore foraging trips in oceanic habitats [16,29,30]. Upon arrival, female elephant seals haul out at a number of sites along the California Coast and offshore islands where they undergo a catastrophic molt [26]. Predation by transient killer whales on grey whale calves and elephant seals increases during spring, and the average group size and number of photo-identified whales are significantly greater during this period than the remainder of the year. This suggests that transients may preferentially use Monterey Bay in the spring to take advantage of the influxes of both prey species as suggested by others [12].

Sightings and encounters with transient killer whales in Monterey Bay decreased dramatically each year at the end of May. The fewest occurrences were in July, when some of the killer whales photo-identified in Monterey Bay were documented along the continental shelf-break and offshore waters of Oregon, Washington, and British Columbia, perhaps following grey whales along their northward migration route [4]. The decrease in sightings in Monterey Bay does not appear to be effort related, as both observer effort and ecotourism in the study area is at its highest in summer. Nor are the seasonal movements we observed likely an artifact of non-systematic surveys given that sighting effort was consistent throughout the year, and analyses of a subset of data consistently recorded by the same experienced naturalists from 2014–2021 yielded the same seasonal patterns in presence. Similar findings have been reported in the coastal waters of Southern British Columbia and Washington State [9].

Some of the whales we observed in Monterey Bay have been seen at other times associating with unidentified transient killer whales in offshore waters and near adjacent deep-sea canyon systems [4]. Movements of whales latitudinally and into offshore waters may provide opportunities to interact with rarely encountered conspecifics within or between subpopulations in order to mate and promote genetic diversity within the population, or to capitalize on seasonally abundant prey. For example, Pacific white-sided dolphins, a prey species of transients in our study, move latitudinally across large ocean basins and into offshore waters during the summer months [31]. Large schools of dolphins in offshore waters during the summer months may provide profitable prey for transient killer whales while in open ocean habitats [32].

The increase in sightings and encounters with transients from September through November coincides with the seasonal northward movements of adult male California sea lions [27] and the arrival of juvenile northern elephant seals [26]. While California sea lions occur throughout the year in Monterey Bay [33], large numbers of the adult males (which are ~3x heavier than adult females) leave breeding sites in the Channel Islands in late July, moving north along the outer coast for the fall and winter [17,34]. Juvenile northern elephant seals arrive to haul out and moult during October off the central coast of California, reaching their highest numbers at haulout sites off Piedras Blancas, Año Nuevo Point, and the Farallon Islands [26]. The presence of male sea lions and juvenile elephant seals most likely influence the seasonal occurrence and movements of transient killer whales during the fall [11].

### Habitat use

In Monterey Bay, the outer coast transient killer whales appeared to concentrate around the high-relief bathymetry of the shelf-break and continental slope of the submarine canyons. The biophysical attributes of these topographic features, specifically depth and seafloor relief, promote complex oceanographic circulation patterns that concentrate the distribution of lower trophic prey (i.e., krill, squid, fish) sought by pinnipeds and small cetaceans that these whales prey upon [35].

Upwelling fronts that form over or near the continental slope transport deep-sea nutrient-rich water to the surface, thereby promoting biological productivity that provides the necessary resources for a diversity of species that inhabit canyon systems [14,35,36]. For instance, California sea lions that haul out along the central coast of California tend to travel and forage along the shelf-break of the Monterey Submarine Canyon, where concentrations of prey species such as northern anchovy (*Engraulis mordax*) and opalescent squid (*Doryteuthis opalescens*) congregate [37–40]. Thus, our increased observations of transient killer whale presence and predation on sea lions in this part of the canyon system likely reflects the biological oceanography associated with upwelling areas (Fig 2B).

Downwelling zones are another important oceanographic factor that likely affects the movements and habitat use of the transients we observed. Downwelling occurs at convergence zones along the shelf-break of submarine canyons, where oxygen rich water and surface biomass in the form of particulate organic matter is transferred to deep ocean waters, increasing the amount of nutrients and resources for mesopelagic fish, demersal fish and cephalopod species [36]. Downwelling zones primarily occur at the rim of submarine canyon systems and along the continental slope, and are known to provide foraging habitat for northern elephant seals and deep diving cetacean species [16,35]. We observed killer whales primarily predating elephant seals over deep continental slope waters exceeding 500 m in depth.

The transient killer whales we observed in Monterey Bay exclusively foraged for prey in open water (>3 km from shore) and were never seen searching for prey along the shore or near pinniped haulout sites as they do in coastal waters of British Columbia, Washington State, and Alaska [1,2,7,41,42]. However, reports from others suggest they may occasionally forage for pinnipeds in shallow nearshore waters of Monterey Bay [12]. This difference in foraging behaviours between inner and outer coast transients may reflect a difference in the culturally transmitted knowledge required to navigate and exploit different habitats. Hunting in the shallow convoluted rocky coastal inland waters of the Pacific Northwest likely comes with a risk of injury or stranding for an uninitiated killer whale, compared to hunting in open ocean habitat where the hunting techniques developed to capture prey that cannot be cornered may not be readily transferable to shallow environments [7].

The transients we observed exhibited two distinctly different foraging behaviours depending on whether they were searching for prey along the shelf break and continental slope of the canyon, or hunting in open water areas unassociated with the bathymetry of the canyon. While in open water, individual killer whales tended to spread out and make erratic movements with long asynchronous surfacing intervals [1,42]. This type of behaviour likely enables a group of whales to search and cover more area, potentially reducing the time spent locating prey and reducing energy expenditure. Species consumed while foraging in open water consisted primarily of small cetaceans (i.e., common dolphins, Pacific white-sided dolphins, Dall’s porpoise).

Transient killer whales use stealth while searching for prey, and primarily employ passive listening to navigate and locate their targets [43,44]. However, it is not known how they navigate the deep bathymetry of the submarine canyon while foraging. One possibility is that the whales can “hear the canyon” by passively listening to water noise that upwells along different bathymetric features (as initially proposed by [45]). This is consistent with the hypothesis that common dolphins inhabiting oceanic waters off California locate deep-sea mounts submerged 2000 m below the surface by passively listening to the structural properties of reflected water noise [46].

### Foraging behaviour and prey handling

Focused research on the foraging behaviour and feeding ecology of transient killer whales began around southern Vancouver Island in the 1980’s [1,6,8], with subsequent studies expanding to the greater coastal waters of British Columbia, Washington State and Alaska [2,9,41,42]. Our observations of these predators in California expand the geographic areas and habitat types studied. Unfortunately, differences in geographic settings, experimental designs, observer effort, and types and definitions of activity states complicates making simple comparisons of activity budgets and other metrics of behaviour between studies. This points to the need to develop a standardized set of field methodologies that can be employed to further the understanding of transient killer whale behavioural ecology.

The outer coast transient killer whales we observed during daylight hours in Monterey Bay spent most of their time foraging—with 84% of their overall activity budget allocated to locating, pursuing, and feeding on prey. This was comparatively higher than the 33–63% of time spent foraging reported in other studies [1,42,47,48]. The greater time spent foraging in Monterey Bay appears to reflect the increased handling time (33% of foraging time) needed to subdue and consume the larger prey present (e.g., grey whale calves and elephant seals) compared with smaller prey (e.g., harbour seals) commonly consumed in the inside coastal waters of British Columbia, Washington State, and Alaska. Most captures and feeding in Monterey Bay were relatively long and occurred at the surface, where we noted coordinated pursuit and feeding on prey. This was particularly evident for California sea lions and grey whale calves that required extensive handling to kill (1–5 hours), and whose large body sizes enabled us to observe prey sharing. This was notably different from the relatively quick handling behaviour of killer whales that prey primarily on harbor seals and small cetaceans that tend to be consumed beneath the surface [1,2].

The prey consumed in Monterey Bay included 9 species of marine mammals and 3 species of seabirds—and involved different prey handling behaviours based on prey size and the group size of the transients present. California sea lions made up nearly half of the observed predation events and contributed regularly throughout the year to the whales’ diet compared to other species. The prominence of California sea lions in the diet of transients likely reflects the abundance of these pinnipeds in our study area. California sea lions are the most abundant marine mammal species in the California Current Ecosystem, with a population of approximately 300,000 [17,49].

Attacks on California sea lions typically involved groups of 5 whales taking turns rushing and ramming sea lions with their bodies, or using their flukes to hit or catapult the sea lions into the air. This behaviour appears to disorient and debilitate the sea lion, so that the whales can safely drag their prey beneath the surface to drown it [1,2,50]. Once a sea lion was dead, whales carried and shared large sections of blubber and tissue that were often passed between group members.

Attacking adult California sea lions can be potentially dangerous for killer whales due to the defensive abilities (sharp teeth) and large body sizes (weighing up to 400 kg for adult males) [1,11,51] of the prey. This was apparent from an analysis of photographs of various transient killer whales that had healed pinniped bite wounds, presumably from sea lions (McInnes, unpublished data). Furthermore, several studies in coastal waters of the Pacific Northwest have shown that extensive hunts by transients on adult California and Steller sea lions typically have a lower success rate than for easier to handle prey such as harbor seals and harbor porpoise [1,42,50].

In contrast to observations of sea lions hunted by killer whales in near-shore coastal waters, all of our observations of transients attacking sea lions in Monterey Bay ended in death. None escaped. This habitat difference in susceptibilities suggests that sea lions have higher probabilities of surviving if they can retreat to kelp beds, navigate through shallow rocky habitats, or haul out on reefs where killer whales are more at risk of being injured [1,7,50]. In contrast, sea lions encountered in deep open pelagic areas have no avenues of escape, where even solitary male killer whales can subdue and kill adult sea lions on their own.

There has been relatively little information on the predatory behaviour of transient killer whales on northern elephant seals in the North Pacific, with most observations coming from single opportunistic predation events [1,11,12], stomach contents from stranded whales [41,50], and historical whaling records of prey remains from killer whales captured in California waters [52,53]. However, recent studies using advanced bio-logging technology and long-term observations have found that male elephant seals that forage along the continental shelf break and slope have a higher risk of predation by white sharks (*Carcharodon carcharias*) and transient killer whales than do female elephant seals that feed further from shore in open oceanic environments [16].

In our case, the elephant seals that were attacked and killed by transients were primarily juvenile and subadult male/adult female elephant seals, and the predation events occurred during the spring and fall over the deep waters of the continental slope of the Monterey Submarine Canyon. Our results further suggest that female elephant seals are most likely opportunistically intercepted and captured as they returned to haulout sites from their offshore foraging trips during spring. The same may also be true for the juvenile seals consumed in the fall.

Adult male elephant seals were never observed as prey. This might be due to seasonal differences in their occurrence and spatial distribution patterns, with males primarily occurring in Monterey Bay in the summer moulting period, and then again during the winter breeding period when transient killer whale occurrence is lowest [16,26]. Male elephant seals are also proficient deep divers that can dive to greater depths for longer periods of time than juvenile and adult female seals, potentially making them more difficult for killer whales and white sharks to capture [16,54]. However, we did receive one anecdotal report from a fisher of a group of 5 killer whales feeding on the carcass of an adult male elephant seal during the month of August in waters offshore of Año Nuevo Island, California (J. McInnes unpublished data).

Both juvenile and adult female elephant seals are presumably profitable prey due to their ease of capture and high energy-rich blubber, and may be an important seasonal prey species for killer whales in Monterey Bay or during periods when the whales are foraging in offshore waters [1,16,32,55]. The importance of elephant seals as prey for mammal-eating killer whales has been extensively studied in the southern hemisphere, with observations of whales primarily feeding on southern elephant seals (*Mirounga leonine*), while using similar predatory and feeding behaviours as we have described [56–58].

The only other pinniped species taken by transient killer whales in Monterey Bay were harbour seals, which made up 5% of observed predation events and did not appear to be a significant prey resource. However, prey handling of these pinnipeds was relatively fast and mainly occurred below the surface, potentially biasing our estimate [1,2]. In contrast to sea lions in California, harbour seals appear to be a major driver of the ecology and behaviour of transients in nearshore regions of British Columbia, Washington State, and Southeastern Alaska [1,2,9]. In the coastal waters off southern Vancouver Island, harbour seals account for 95% of observed predation events [1] and 67% of the diet of transients in British Columbia [50]. Harbour seals are less abundant along the central coast of California compared to other species (i.e., California sea lions, northern elephant seals) [17], and primarily occur at haulout sites in harbours, sloughs, mudflats, and rocky reefs—areas where we did not encounter killer whales [59,60].

Grey whale calves were the second most commonly observed prey species and appear to be a seasonally important resource for transient killer whales, with observations of attacks occurring off the central coast of California dating back to the 1960’s [11,12,22,61–65]. [11,12,22,61–65]. This is consistent with other observations of predation events during whale watching ecotours that reported 35% involved grey whale calves—the second most important prey species in Monterey Bay [12]. In addition, indirect evidence of killer whale predation events has also been documented during postmortem examination of wounds left on stranded grey whale juvenile and calf carcasses along the California and Oregon coasts [11,66].

Grey whale calves also appear to make up a important seasonal component of the diet of transient killer whales that occur much further north in Alaska and the Bering Sea [67,68]. In the transboundary continental shelf waters of the Aleutian Islands, 197 photo-identified transient killer whales have been observed over time using the shallow straits of False and Unimak Pass in the months of May and June to ambush grey whales migrating from the North Pacific to the Bering Sea [24,68,69].

Preying on exceedingly large species, such as grey whale calves accompanied by defensive mothers, comes with the risk of injury to the predator, and requires expending considerable time and energy to handle and subdue calves [22,65]. The rewards of net energy gained by killing a grey whale calf must offset the associated risks of the hunt [70]. This may explain why we consistently observed large groups of transient killer whales forming in Monterey Bay during the spring—with most hunts on grey whale calves involving 15 or more whales, suggesting that the probability of successfully killing a grey whale calf increases with killer whale group size. However, bigger group sizes comes at a cost of having to split the prey between more members of a group. Large groups of whales may be better able to overcome grey whale mothers to access their calves (hunting hypothesis), or large groups of transients may have a greater range of collective skills or experiences needed to take large prey (skill-pool effect) [22,71,72]. Large group size may also enable killer whales to protect or defend carcasses from scavengers such as sharks during prolonged feeding periods on large prey [72,73]. We observed blue sharks on two occasions circling nearby grey whale calf carcasses that whales were consuming.

As social predators, beneficial social interactions may occur between killer whales post-predation events, with bigger carcasses providing more animals with the time and opportunity to socialize [74]. Most grey whale calf predations we observed involved the arrival of new groups of transient killer whales into the kill area from offshore regions, as has been noted by others [12]. In our case, this occurred on 15 of the 19 successful hunts on grey whale calves, when new whales either joined the initial group to finish off the calf, or arrived in time to share in feeding on the carcass. Similar behaviour has been observed elsewhere during predation events on sperm whales and minke whales [32,72,75]. Intergroup prey sharing during the grey whale migratory period may potentially provide mating and social opportunities between whales that would otherwise be dispersed in other regions of the coast (see Seasonality).

Female transient killer whales appeared to be the most active and effective group members during predations on grey whale calves, which is consistent with predation events observed in Monterey Bay and elsewhere in the North Pacific [12,65,67]. However, adult males often helped to subdue the calves. Surprisingly, transient killer whale calves as young as 6 months also participated in the hunts and were frequently observed surfacing with females in close association to grey whale mother-calf pairs.

As matrilineal leaders, female killer whales may have extensive knowledge of methods of handling grey whale calves. Observations of particular photo-identified females attacking calves date back to the 1990’s (McInnes unpublished data). Predatory behaviours required to subdue grey whale calves are likely complex and risky, and may require a long and steep learning curve for calves and juveniles that require time to obtain knowledge by observing and participating in hunts with experienced adult females. This is consistent with the high parental investment made in developing the skills of juvenile killer whales in the Crozet Islands to successfully capture southern elephant seals using the high-risk behaviour of intentional stranding [56].

Transient killer whales employ distinct predatory behaviours (i.e., separation and submergence) to subdue and kill grey whale calves. They strategically herd and bite the pectoral fins and flukes of the calves to impede movements, and enable others to work together to separate the calf from its mother [11,22,65]. Body ramming appears to be an effective means to debilitate calves, with individual transients rushing in and striking the sides and head region of the calf. During 3 predation events, we noted adult male killer whales delivering devastating blows to the head regions of grey whale calves.

A potentially interesting observation was the presence of blood emanating from the mouths of calves during 4 predation events. It appeared that the killer whales targeted the head to try and break the calf’s jaw, making it difficult for the calf to surface and breath, and allowing the transients to access the submandibular sac to feed on the tongue. Shortly after such attack behaviour, we observed the killer whales accessing the opened mouth and feeding on the tongue. One calf washed ashore the following day with a broken lower jaw and missing tongue [11]. Transients killed grey whale calves primarily by leaping on top of and forcing the calves beneath the surface.

The extensive time spent feeding on grey whale calves (from 1–12 hours) presumably reflects the considerable mass of food they represent (ca. grey whale calf 900–1000 kg TT) [76]. In addition to feeding on the submandibular sac and tongue, killer whales also removed and fed on chunks of blubber from other parts of the grey whale calves’ bodies, suggesting that other areas of the carcass are also utilized [22,24,65,75]. Similar feeding utilization of grey whale carcasses has been documented by transient killer whales in the Aleutian Islands, Alaska. In this region, killer whales can prolong feeding by storing carcasses in shallow water, and returning to them to remove chunks of blubber and muscle tissue [24].

### Future research

While our study increases knowledge of the behavioural ecology, diet, and seasonal occurrence patterns of outer coast transient killer whales that exploit submarine canyon systems, it is ultimately a small piece of a much larger ecological puzzle. The Monterey Submarine Canyon is situated in the middle of a large bathymetrically complex continental shelf that is interrupted by a series of different canyons that extend into the open ocean. As an apex predator with a largely continuous geographical distribution, transient killer whales likely use multiple submarine canyon systems throughout California waters. For this reason, comprehensive and collaborative offshore surveys using advanced biologging technology (satellite tags) and passive acoustic monitoring would significantly augment knowledge of the spatial and temporal distribution patterns of transient killer whales in less accessible offshore areas. Additional localized studies of transient killer whales using digital archival tags (Dtags) would also provide more detailed information on how these whales use the Monterey Submarine Canyon system, including diving behaviour, subsurface prey handling, and movement patterns in relation to specific topographic features of the canyon [77]. Such studies would significantly contribute to management plans concerning the habitat needs of transient killer whales within the Monterey Bay National Marine Sanctuary as they relate to marine and environmental laws in the state of California.

## Materials and methods

### Study area

Our study of outer coast transient killer whales was centered within Monterey Bay (~36.80° N, 121.94° W), over a ~1200 km^2^ area (Fig 1) that is completely open to the Pacific Ocean and is located within the Monterey Bay National Marine Sanctuary (MBNMS) [13]. The largest bathymetric feature of the study area is the Monterey Submarine Canyon that divides the bay in half, with waters as deep as ~2000 m over the central channel of the main canyon [78]. The head of the central channel begins 0.5 km off Moss Landing, and the widest point of the canyon is 12 km across. Two secondary canyons known as the Soquel and Carmel Canyons extend from the main central channel. The central channel of the Monterey Submarine Canyon continues 470 km beyond the bay into offshore waters of the Monterey Deep Sea Fan and abyssal plains [79].

Monterey Bay is located along the central coast of California—a highly productive marine region [80] that is supported by the nutrient rich eastern transboundary California Current that flows southward along the west coast of North America. The central coast of California has a narrow continental shelf (<1–50 km) that results in oceanic waters and complex bathymetric features such as deep pelagic submarine canyons occurring close to shore [35,78,80,81]. In addition, complex ocean circulation patterns driven locally by seasonal coastal upwelling fuel productivity—producing a multifaceted marine food web and an important foraging area for predators such as marine mammals [14,31,82,83].

### Seasonal occurrence data

The photographs and sightings reports we used to investigate the seasonal occurrence patterns of transient killer whales in Monterey Bay were compiled on a continual basis from an extensive network of whale watch naturalists throughout each year from 2014–2021. Sightings data were excluded from 2020 (because COVID-19 limited whale watching trips) and from 2015 (because few sightings were consistently recorded)—resulting in 6 years of data. Data were collected more consistently from whale watch sightings than from marine mammal research surveys, and were therefore used to analyze differences in monthly and seasonal occurrences and group sizes of transient killer whales (Table 3). The minimal data collected from each sighting included the date, time (24-hour clock), location (latitude and longitude), group size, photographs, identification of whales, and observations of predation events.

**Table 3:** Description of the two main sources of data collection that outline key sampling parameters used for the seasonal, geospatial, and behavioural analyses in this study.

Data source	Years	Description	Number and type of observations	Type of analysis
Marine mammal surveys	2006–2018 (Primarily March-November)	Dedicated small vessel marine mammal surveys. Extensive behavioural data collected during focal follows.	100 encounters	• Activity budget • Behavioural classification • Geospatial analysis • Kill rate • Photo-identification • Prey species identification
Whale watch ecotour reports	2014–2021 (year-round)	Continuous sighting reports from whale watch ecotour companies. Discrete observations of predation events, group size, and location information.	161 sightings	• Seasonal occurrence • Seasonal comparison of group size • Geospatial analysis • Photo-identification • Prey species identification

From 2014–2021, the commercial whale watch industry in Monterey Bay was composed of approximately 10 companies that operated 19 different vessels throughout the year. During the primary season (April through October) companies ran 2 to 3 trips per day that lasted 2 to 4 hours. In addition, three companies offered extended specialized killer whale ecotours in the months of April and May that were 6 to 9 hours long. During the winter season (November through March), companies ran 1 to 2 trips per day, each lasting 2 to 3 hours. When killer whales were encountered, ecotour operators typically observed them for 30 minutes to 3 hours (D. Frank pers. comm. Discovery Whale Watch, 2022).

Using community acquired sightings data can result in spatial and temporal biases due to opportunistic observation effort. While accounting for such observation-related biases was beyond the scope of our study, other studies in the coastal waters of the Pacific Northwest have demonstrated that such data can be a reliable and effective means to determine the spatial distribution and seasonal occurrence patterns of killer whales—assuming that georeferenced locations, correct photo-identification, and limitations of the study are recognized [9,84,85].

For our study, we limited the geographic scope to sightings within Monterey Bay that had georeferenced locations and were accompanied with photographs from experienced naturalists. We analysed occurrences to determine how frequently transient killer whales visited and seasonally used Monterey Bay [9]. In doing so, we **1)** confirmed the photo-identification of each matriline and individual whale in a particular georeferenced sighting; **2)** examined the number of concurrent days that a particular matriline and individual was reported in Monterey Bay; and **3)** reduced the number of sightings to a measure of occurrence—defined as being present on a given day. Through this preliminary analysis, we found that the largest number of concurrent days that an individual or group was observed was 4 days. We therefore assumed that specific transient matrilines and individual whales had likely left the Monterey Bay area if they were not encountered again within this 4-day interval. Subsequent reports of the same group or individual outside a 4-day window were considered a separate occurrence (Table 4). We then used the cumulative occurrence to analyze differences in the occurrences of transient killer whales among months and seasons.

**Table 4:** The number of consecutive days that photo-identified transient killer whales were observed in Monterey Bay, California (2014–2021).

Number of consecutive days	Frequency
1	319
2	38
3	6
4	5
5	0

Previous studies have reported increased sightings of transient killer whales during the northbound migratory movements of female grey whales with newborn calves along the central coast of California [11,12,64,65,86]. To explore this ecological interaction, we compared the seasonal occurrence patterns of photo-identified individual killer whales with census counts of grey whale mother-calf pairs collected from 2014–2021 (2015 and 2020 excluded) to provide a visual representation of seasonal co-occurrence for both species. Grey whale mother-calf count data are collected by researchers at the National Oceanic and Atmospheric Administrations (NOAA) Southwest Fisheries Science Center (SWFSC). Dedicated grey whale censuses are conducted annually between late March and early June from shore-based counts off Piedras Blancas Light Station, ~160 km south of Monterey Bay (Fig 10). By late June, counts are stopped because grey whale mothers with calves are far north of the census study area [18–20]. From these data, two ecological seasons were defined—March-May (grey whale northward migratory period) and June-February (no female-calf pairs present).

### Geospatial analysis

Data from whale watch sightings and marine mammal surveys with transient killer whales (2006–2021) (Table 3) were analyzed using ArcGIS Pro (ESRI, Redlands California, version 3.0.4) to **1)** describe the general geographic spread of observations in Monterey Bay, **2)** visualize specific aspects of behaviours in relation to bathymetric features recorded during focal follows, **3)** plot the locations of predation events of different species of prey, and **4)** analyze the density of observations in relation to specific physiographic features of the Monterey Submarine Canyon.

Bathymetric features, canyon contours, and observations were projected onto the appropriate UTM zone (WGS 1984). Bathymetric data and water depths for Monterey Bay were extracted using NOAA’s National Centers for Environmental Information (NCEI) 1/3 arcsecond (10 m) Digital Elevation Model (DEM). Based on the historical definition for the spatial characteristics of the continental shelf system, we defined the continental shelf to extend out from shore to the continental shelf break at the 200 m isobath and the upper slope to occur at the 500 m isobath [78,81].

To investigate the density of observations, we used a Kernel density tool to visualize areas of high to low spatial density of transient killer whales in Monterey Bay. Kernel density estimates represent the 2-dimensional relative frequency, and were estimated for observations according to:

$$Density=\frac{1}{{\left(radius\right)}^{2}}\sum_{i=1}^{n}\left[\frac{3}{n}\times {pop}_{i}{\left(1-{\left(\frac{{dist}_{i}}{radius}\right)}^{2}\right)}^{2}\right]$$ (1)

$$For\;{dist}_{i}<radius$$

where *i* = 1 …, n are the input points, *pop*_*i*_ is the population field value of point *i*, and *dist*_*i*_ is the distance between point *i* and the (x, y) georeferenced location of the observation. The Kernel density tool provided a predictive gradient across the study area, enabling us to visualize areas of higher habitat use.

### Behavioural data

From 2006 to 2018, three to seven people aboard small research vessels (4 to 8 m) recorded behavioural observations of transient killer whales during marine mammal surveys. While surveys were conducted throughout the year, effort was highest in the months of March to November. We defined an encounter as a period greater than 10 minutes, where all individual whales in a group could be photo-identified and were close enough to the research vessel that the observers were able to classify behaviours, and prey species during predation events. We followed whales at a relatively far distance (100 m) to avoid disrupting their normal behaviour as much as possible. When a confirmed predation event was in process, we attempted to maneuver the research vessel within 50 m to record the species of prey and prey handling techniques used by the whales.

At the onset of an encounter, a primary observer voice recorded or transcribed data while two other observers photographed the whales using high quality digital SLR cameras with telephoto lenses. Individual whales were identified using at least two distinctive, unique markings, including notches or nicks on the dorsal fin, scarring on the saddle patch, and the shape of the dorsal fin and saddle patch [87,88].

The transient killer whales we encountered in California waters are assigned alphanumeric designations (e.g., OCT030), and confirmation of group membership was made by comparing photographs using published catalogs [4,11] based on the OCT (outer coast transient) alphanumeric system. We thus used photo-identification methodology to obtain overall counts and to collate information on individual group life histories.

Transient killer whales live in fluid matrilineal groups comprising a mother and her associated offspring—with dispersal of offspring occurring for some adult males and most females at the onset of sexual maturity [74,89]. Due to their fluid social structure, we defined a matriline as a mother and all her offspring that have not been known to have dispersed. Single adult males were defined as their own group if they spent >50% of their time not associated with any particular known matriline. During an encounter, all visible surface behaviours of a group were recorded simultaneously using focal group sampling methodology [1,90]. The duration of different behaviours was determined by noting the time at which a change in behavioural state occurred. Focal group sampling was deemed an appropriate means to collect our data because **1)** transient killer whale group sizes were predominantly small, **2)** whales demonstrated highly coordinated behaviours that were typically exhibited by all members of a group, and **3)** the sampling techniques were tailored to resemble previous behavioural studies of transient killer whales for comparative analysis [1].

The data we collected included the date, time (24-hour clock), georeferenced location (latitude and longitude), identity of individual whales, group size, direction of travel, dive durations, estimated distance between individual whales, and active surface behaviours (i.e., spy hop, breaching, tail lob, porpoising, prey capture techniques). Environmental conditions such as wind and wave height (Beaufort scale), visibility, water depth (m) and percentage of cloud cover were also recorded. Throughout an encounter, we continuously assessed these parameters, and recorded location data using a global positioning system (GPS) every 5 minutes to track the movement patterns exhibited by a group of whales while engaged in different behaviours until an encounter was terminated. Reasons for ending encounters included adverse weather conditions, fuel constraints, lighting conditions, and loss of whales.

Predation events were noted when transient killer whales were actively pursuing or feeding on marine mammal prey, and included observations of prey or prey parts in the mouths of whales or remains of prey were visible at the sea surface (i.e., bits of blubber, blood, muscle tissue, internal organs, oil slick). The times from when the whales encountered prey until prey death (prey pursuit)—and from death to consumption or abandonment of the prey carcass (feeding)—were also recorded. In addition, we recorded the time, location, group size, prey species, estimated prey size, number of other marine mammal species in the general area, and observations of harassment or non-predatory interactions. Only prey that could be identified to species were included in our analyses.

Activity states were partially derived from definitions of transient killer whale behaviours by [1] and modified based on [91] (Table 5). To create an activity budget, the time each matrilineal group spent in each behaviour was summed and divided by the length of that observation period [90]. The total time in each behaviour was then divided by the total time spent observing whales. All behavioural data were analyzed using the R-statistical package behaviouR [92] in R software v. 4.1 [93].

**Table 5:** Categories used to quantify the behaviours of transient killer whales.

Category	Description
Travelling	Travel continuous and in a consistent direction at speeds greater than 5 km/hr. Individuals travelling within a few body lengths of each other; respirations synchronous; prey is occasionally caught and killed. Difficult to distinguish travelling from foraging.
Open Water foraging	Long asynchronous dives (>8 minutes); movement patterns variable with whales zigzagging; spread out greater than 5 body lengths apart and up to 1 km; typically involves predation on porpoises, dolphins, and pinnipeds.
Shelf break/canyon foraging	Following the contours of the continental shelf break and along the edge of deep-sea canyons; long synchronous dives (5–7 minutes); whales typically less than 6 body lengths apart; typically involves predation on grey whale calves.
Prey pursuit	Predatory interactions with prey: high-speed chases of short or long duration; prey rammed with whale’s rostrum or hit with pectoral fin or flukes; prey thrown into the air or dragged beneath the surface.
Feeding	Prey or parts of prey visible in the whale’s mouth or at the surface; individuals milling and diving in same location; blood and oil slick (animal fat) at the surface; birds hovering or picking up tissue at the surface.
Socializing	Interactive movements not associated with prey capture. Typically involves percussive behaviour (e.g., breaches, spyhops, flipper slapping, tail slapping).
Resting	Respirations synchronous; whales less than 1 body length apart; movement consistent but slow with little headway against a current, hanging motionless at the surface.

To investigate predatory interactions between transient killer whales with different prey species, we calculated a kill rate [2] by dividing the total number of hours spent observing them by the number of kills recorded for the two most commonly observed prey species—California sea lions and grey whale calves. The remaining prey species were pooled. We then multiplied the average number of kills observed per hour by 24 hours, and divided it by their average group size, to obtain a daily kill rate per individual whale based on:

$$Kill\;rate=\frac{number\;of\;kills\;per\;prey\;species}{number\;of\;hours\;followed}\times 24÷average\;group\;size$$ (2)

## Supporting information

S1 Table Transient killer whale seasonal occurrence information. Date, season, and group size of transient killer whales sighted during whale watch ecotours in Monterey Bay, California.
(XLSX)

S2 Table Transient killer whale focal follow data. Behavioural data collected during research vessel focal follows. Data include date, time, geospatial location (latitude and longitude), number of photo-identified whales, behaviour designation, sub behaviour, and prey species.
(XLSX)

S3 Table Transient killer whale diet effort and sample summary. Information on the date, geospatial location (latitude and longitude), prey species, and group size of transient killer whales when foraging.
(XLSX)

S4 Table Seasonal differences in group size. Table providing information on date, season, and group size, transient killer whales collected from whale watch ecotours.
(XLSX)
